# Volvulus du sigmoide necrosé du postpartum

**Published:** 2012-07-04

**Authors:** Ihsane Moussaid, Wafaa Haddad, Said Salmi, Smaïl Elyoussoufi, Mohamed Miguil, Sihame Serbouti, Houssine Boufettal, Naima Samouh, Saad Berrada

**Affiliations:** 1 Service d'anesthésie réanimation de la maternité CHU Ibn Rochd Casablanca, Maroc; 2 Service de gynécologie-obstétrique maternité Lalla Meriem CHU Ibnou Rochd, Casablanca, Maroc; 3 Service des urgences chirurgicales CHU Ibnou Rochd Casablanca, Maroc

**Keywords:** Postpartum, necrose, volvulus, chirurgie, sigmoide, Postpartum, necrosis, volvulus, surgery, sigmoid

## Abstract

Le volvulus du sigmoïde compliquant la grossesse est une complication rare avec moins de 80 cas rapportés dans la littérature. Nous rapportons un cas de volvulus du sigmoïde compliquant les suites de couches. Une primigeste de 29-ans s'est présenté avec une douleur abdominale à J3 du post-partum. Une laparotomie exploratrice a révélé un volvulus, nécrosé, du côlon sigmoïde très distendu. Dans notre cas. Le Volvulus du sigmoïde est susceptible d'avoir été précipité par le changement rapide de la taille de l'utérus après l'accouchement. La mobilité colique associée à une distorsion du côlon sigmoïde, doit être gardé à l'esprit chez les patients qui présentent des douleurs abdominales lors du post-partum. Cet article se propose à travers un cas clinique de revoir les données de la littérature et d'engager les principes généraux de prise en charge et de traitement.

## Introduction

Le volvulus est une complication rare de la grossesse, nécessitant une intervention chirurgicale immédiate. Il serait la deuxième cause après les occlusions sur brides, environ 80 cas ont été décrits dans la littérature mondiale. Son diagnostic reste difficile et son pronostic essentiellement conditionné par la précocité du traitement [1]. Nous rapportons l'observation d'une patiente, admise pour syndrome occlusif en post partum d'un accouchement prématuré de 30 semaines d'aménorrhée.

## Patient et observation

**Figure 1:**
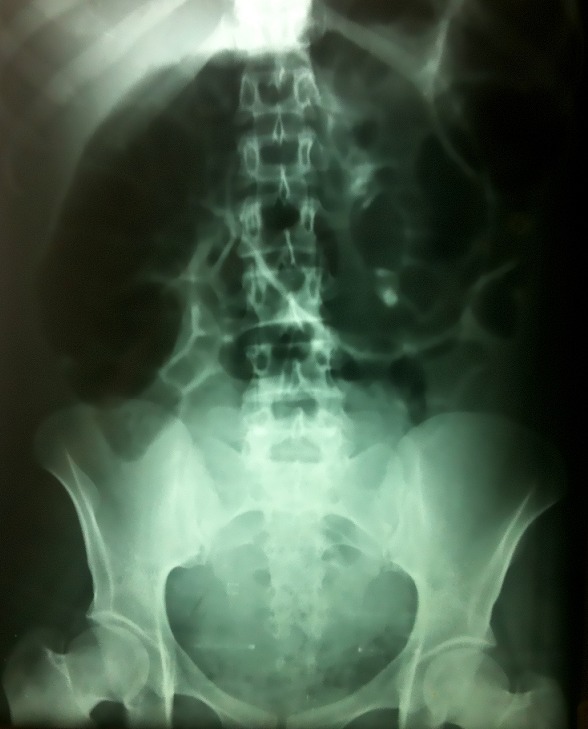
Abdomen sans préparation (ASP) objectivant une distension colique très importante avec un arceau gazeux à double jambage

**Figure 2:**
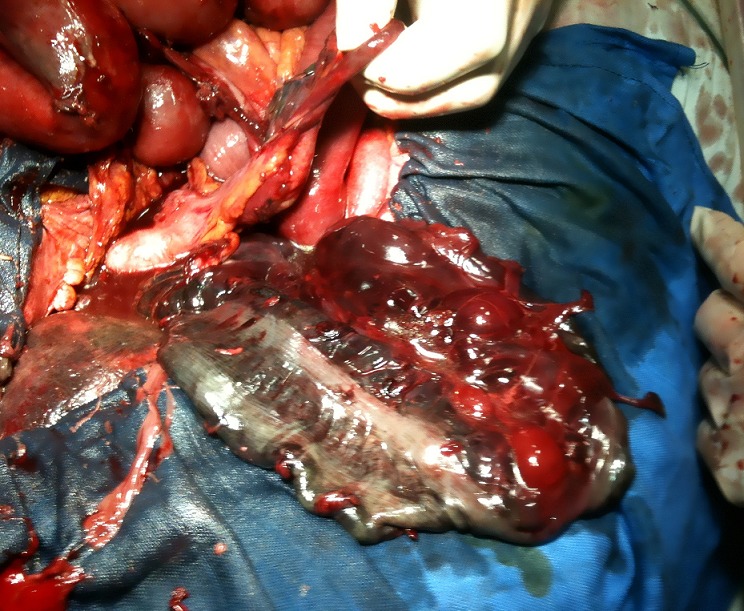
Exploration chirurgicale montrant un volvulus du sigmoïde nécrosé

**Figure 3:**
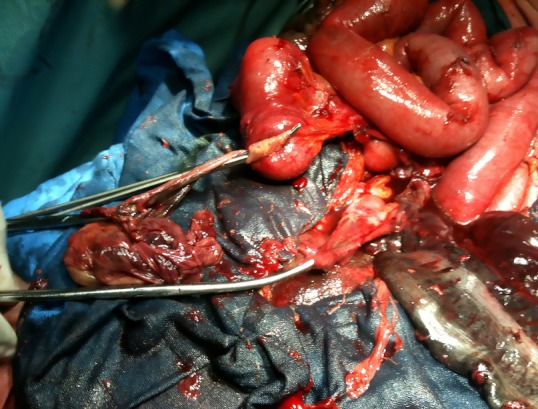
Une sigmoïdectomie emportant le sigmoïde et l'anse iléale nécrosés a été réalisé

Mme HR, âgée de 29 ans, sans antécédents pathologiques, transféré d'une maternité niveau II à j2 d'un accouchement prématuré donnant naissance à nouveau-né de sexe masculin Apgar 8/10 sur une grossesse estimé à 30 semaines d'aménorrhée, pour douleurs abdominales diffuses évoluant depuis deux jours, associées à des vomissements et arrêt des matières et des gaz. L'examen physique a révélé un état général altéré avec une fièvre à 38,5 °C, une tension artérielle à 90/60 mm Hg et un pouls à 88 battement/min. L'examen abdominal a objectivé un météorisme important de la région épigastrique avec une défense abdominale généralisée. Au toucher rectal, l'ampoule rectale était vide. L'examen gynécologique, a trouvé un col ouvert à un doigt, utérus difficile à apprécié vu la distension abdominale. L'abdomen sans préparation (ASP) a objectivé une distension colique très importante avec un arceau gazeux à double jambage (Figure 1). L’échographie abdominopelvienne a montré une importante distension digestive avec stase stercorale associée à un épanchement intrapéritonéal. Le bilan biologique a montré une hyperleucocytose à 17 610 éléments/mm3 (éléments/mm3, normale inférieure à 10 000 éléments/mm3). Le diagnostic de péritonite sur volvulus du sigmoïde a été retenu, et une exploration chirurgicale s'est imposé. Après une laparotomie médiane et une aspiration de 1000 ml d'un liquide brunâtre fétide, l'exploration a mis en évidence un dolichocôlon compliqué par un volvulus du sigmoïde nécrosé (Figure 2), atteinte de l'ilion, avec présence de quelques fausses membranes. Nous avons réalisé une sigmoïdectomie emportant le sigmoïde nécrosée et l'anse iléale nécrosé (Figure 3), iléostomie droite et colostomie gauche (intervention d'Hartmann).

## Discussion

Le volvulus du sigmoïde est généralement rapporté chez des patients affaiblis âgés ou souffrant de constipation chronique. Une incidence élevée signalée en Afrique a été attribué à l'alimentation riche en fibres végétales chez cette population [2].

Les causes courantes d'occlusion intestinale au cours de la grossesse comprennent les adhérences, les volvulus, l'invagination intestinale, la hernie et l′appendicite. Le volvulus du côlon sigmoïde est la cause la plus fréquente d′occlusion intestinale compliquant la grossesse, il représente jusqu′à 44 pour cent des cas [2]. Une autre étude a montré que dans environ 25% des cas, le volvulus affecte généralement le gros intestin [3]. Dans notre cas, il n′y avait aucun antécédent d'intervention chirurgicale et il n′y avait aucune adhérence au cours de l'exploration, l'occlusion était secondaire à un volvulus.

Le volvulus du sigmoïde est très rare chez la femme non gravide en âge de procréer; il se produit le plus souvent chez la femme enceinte dans le troisième trimestre. Harar et Harar ont émis l′hypothèse que cela peut être dû à la taille croissante de l′utérus élevant un colon sigmoïde mobile du bassin et produire une occlusion partielle, soit en raison de la pression ou de la coudure de l′intestin [4].

Le diagnostic d′occlusion intestinale pendant la grossesse est souvent retardé car les symptômes miment les autres complications de la grossesse. Les signes classiques d'occlusion digestive telle que les vomissements, la distension abdominale et la constipation, peuvent être absente. Un examen échographique peut aider au diagnostic différentiel. La radiographie de l'abdomen sans préparation montre des aspects typique de l′obstruction dans 91% des cas. La dose usuelle de 0,001 Gy par examen, même répétés pour le suivi des patientes ayant une occlusion intestinale suspectée, comporte un risque négligeable pour le fœtus lors du troisième trimestre [5].

Le traitement de l′occlusion intestinale chez les femmes enceintes est similaire à celui des femmes non enceintes. La chirurgie est la base du traitement et doit être réalisée par incision verticale médiane. Au troisième trimestre, si l'exposition intestinale ne peut être obtenue, une césarienne doit être réalisée. L′ensemble de l'intestin devraient être examinées, la viabilité doit être évaluée avec soin et la réalisation de résection segmentaire avec ou sans anastomose est souvent nécessaire [6].

Lorsque l′occlusion intestinale complique la grossesse, le pronostic à la fois de la mère et le fœtus devient réservé. Dans une série de 66 femmes enceintes présentant une occlusion intestinale, 23% ayant requis une résection intestinale avec un taux de mortalité fœtale de 26% et quatre décès maternels [6]. Dans une brève communication par Sascha Dua, une parturiente a présenté 3 jours après l′accouchement un volvulus du sigmoïde semblable à notre cas. Le volvulus du sigmoïde est susceptible avoir été précipité par la mobilité colique associée à une distorsion du côlon sigmoïde, et aussi par l′involution rapide de l′utérus [7].

Diagnostiquer la cause d′un abdomen aigu est difficile dans le post-partum immédiat. L'augmentation de volume de l′abdomen et la difficulté d′obtenir des signes abdominaux (en raison de la perte de tonus de la paroi abdominale) peut masquer les signes de péritonite.

## Conclusion

L'occlusion digestive complique rarement une grossesse; cependant, elle engage systématiquement le pronostic vital de la mère et de l'enfant. Le retard thérapeutique et diagnostic sont les principaux facteurs de morbidité et mortalité maternelle et fœtale. La prise en charge doit être multidisciplinaire par un radiologue, un obstétricien, un réanimateur et un chirurgien.
